# Repeated double cross-validation applied to the PCA-LDA classification of SERS spectra: a case study with serum samples from hepatocellular carcinoma patients

**DOI:** 10.1007/s00216-020-03093-7

**Published:** 2020-12-08

**Authors:** Elisa Gurian, Alessia Di Silvestre, Elisa Mitri, Devis Pascut, Claudio Tiribelli, Mauro Giuffrè, Lory Saveria Crocè, Valter Sergo, Alois Bonifacio

**Affiliations:** 1 Raman Spectroscopy Lab, Dipartimento di Ingegneria e Architettura (DIA), University of Trieste, via Valerio 6, 34127 Trieste, TS Italy; 2 Fondazione Italiana Fegato – ONLUS, Area Science Park, SS14, km163.5, 34149, Basovizza, Trieste, TS Italy; 3 Department of Medical Sciences, University of Trieste, Strada di Fiume, 447, 34129 Trieste, Italy; 4 Faculty of Health Sciences, University of Macau, Macau, SAR People’s Republic of China

**Keywords:** SERS, Double cross-validation, PCA-LDA, Serum, Hepatocellular carcinoma

## Abstract

Intense label-free surface-enhanced Raman scattering (SERS) spectra of serum samples were rapidly obtained on Ag plasmonic paper substrates upon 785 nm excitation. Spectra from the hepatocellular carcinoma (HCC) patients showed consistent differences with respect to those of the control group. In particular, uric acid was found to be relatively more abundant in patients, while hypoxanthine, ergothioneine, and glutathione were found as relatively more abundant in the control group. A repeated double cross-validation (RDCV) strategy was applied to optimize and validate principal component analysis-linear discriminant analysis (PCA-LDA) models. An analysis of the RDCV results indicated that a PCA-LDA model using up to the first four principal components has a good classification performance (average accuracy was 81%). The analysis also allowed confidence intervals to be calculated for the figures of merit, and the principal components used by the LDA to be interpreted in terms of metabolites, confirming that bands of uric acid, hypoxanthine, ergothioneine, and glutathione were indeed used by the PCA-LDA algorithm to classify the spectra.

**Supplementary Information:**

The online version contains supplementary material available at 10.1007/s00216-020-03093-7.

## Introduction

Surface-enhanced Raman scattering (SERS) spectroscopy is an analytical technique based on the inelastic scattering of a laser by analytes adsorbed on nanostructured metal surfaces with adequate plasmonic properties [1, 2]. As for normal Raman spectroscopy, bands in SERS spectra are related to the different vibrational modes of the analyte molecules. Different molecular structures will yield different spectra, making vibrational spectroscopies as Raman and SERS very structure-specific. However, SERS benefits from a much greater sensitivity than Raman, due to the intensity enhancement granted by its interaction with the plasmonic surface. These characteristics, together with the availability of relatively inexpensive and portable instrumentation, as well as a fast analytical response, make SERS extremely appealing for bioanalytical applications, many of which are listed in recent reviews [3, 4].

One of the simplest approach used when applying SERS to bioanalysis, usually referred to as label-free SERS, consists of putting a biofluid containing the analyte or a mixture of analytes in contact with a nanostructured metal surface (such as metal nanoparticles) for direct detection of the target molecule(s). While in some cases a specific analyte is sought, in many cases, especially when developing a diagnostic method, an untargeted approach is adopted. By using this strategy, the rich biochemical complexity of biofluids such as blood plasma or serum is explored, and not just one but several metabolites are considered in a multi-marker approach to diagnosis. Thus, in a study where label-free SERS is used to characterize biofluid samples for diagnostic or prognostic purposes, spectra become a sort of metabolic fingerprints, in which bands originate from those narrow subset of metabolites with a higher affinity for the nanostructured metal surface [5].

Label-free SERS of biofluids such as plasma, serum, urine, or saliva is rapidly emerging as a promising method for the diagnosis of several pathologies [3–5], especially by using multivariate data analysis and predictive modelling methods to fully exploit the intrinsic multivariate information present in the spectral dataset. By using multivariate prediction algorithms [6–8], even what are usually considered extremely small spectral differences can be exploited for classification purposes. However, multivariate methods are a two-edged sword, and while being extremely powerful tools to exploit the information contained in SERS spectra, they should be carefully validated and the results correctly presented. To avoid overfitting, and thus a gross over-estimation of the classification performance of a method, a careful approach should be adopted when trying to optimize and validate a model. Another issue in predictive models is the estimation of the uncertainties of figures of merit (FOM, also addressed as “quality performance metrics”) such as accuracy, sensitivity, specificity, NPV, PPV, and AUC, often used [9] to express the performance of a classification model. The uncertainty about a model performance can be conveyed by specifying confidence intervals for FOM. However, such confidence intervals cannot be derived from a single model, but require an adequate number of different models.

Among the different strategies available for optimization and assessment of models, the repeated double cross-validation [6, 10] (RDCV, see Methods and Discussion for details) has one advantage it automatically optimizes model parameters, thus avoiding arbitrary choices by researchers, while keeping train and test data sets for optimization and validation well separated. These features help to minimize the possibility of overfitting. Moreover, the repeated cross-validation generates many different models that can be used to calculate confidence intervals for FOM.

This paper aims to apply RDCV for classification, using a “principal component analysis - linear discriminant analysis” approach (PCA-LDA [6], see Methods and Discussion for details) on a label-free SERS dataset. RDCV has been originally proposed and used for regression [10], and although a number of studies applied this approach to classification as well on several types of spectroscopic data [11–15], to our knowledge, it has never been applied with this purpose to SERS data. As a case study to assess the use of RDCV, we use a dataset of label-free SERS spectra of serum of two groups of subjects: patients with hepatocellular carcinoma (HCC) and a control group.

The focus on HCC derives from the evidence that early diagnosis for this cancer still represents an unmet clinical need. HCC is the most common type of primary liver cancer, represents the seventh most frequent cancer and the fourth leading cause of cancer-related death worldwide in 2018 [16]. The late diagnosis has a negative impact on patients’ life expectancy since it lowers the chances of effective treatment options. HCC is the only cancer diagnosed through imaging techniques without the need for histological confirmation. However, imaging techniques have some limitations in terms of sensitivity, costs, and patient’s compliance. Short-term surveillance with these techniques is still considered not clinically efficient and cost-effective. New non-invasive tools are thus needed to for early HCC detection, and label-free SERS of serum or other biofluids might be a viable candidate.

## Materials and methods

### Materials and chemicals

All chemicals used for the SERS substrate fabrication were purchased from Merck and used as received. Pure cellulose qualitative filter paper (grade 410, 2 μm average pore size) was purchased from VWR International Srl (Milano, IT). Ultrapure water (Milli-Q) was used for preparing all solutions.

### Human serum samples

Fasting blood samples were collected at time of diagnosis from 72 consecutive male subjects with HCC referring to the Liver Center of the University Hospital of Trieste (Italy) and from 72 consecutive healthy blood donors recruited in 2018 at the Transfusion Clinic of the University Hospital of Trieste (Italy) (Table 1, and Table S1 in the Supplementary Information (ESM)). All the patients provided written informed consent and patient anonymity has been preserved. The investigation was conducted according to the principles expressed in the Declaration of Helsinki. The study was approved by the regional ethical committee (Comitato Etico Regionale Unico del Friuli Venezia Giulia, Prot. No. 2018 Os-008-ASUITS, CINECA no. 2225). HCC was diagnosed according to the EASL criteria and staged according to the Barcelona Clinic Liver Cancer (BCLC) [17].

**Table 1:** Characteristics of the study populations. Age expressed as median (1st quartile–3rd quartile). For more characteristics, see Table S1 in ESM

	Number of samples	Age
Controls (CTR)	72	56 (52–60)
Hepatocellular c. (H0T)	72	69 (64–74)
TOTAL	144	61 (55–69)

### Sample collection and storage

Serum samples were obtained from 6 mL of whole blood collected in Vacuette® serum separating tubes (Greiner Bio-One International GmbH, Kremsmünster, Austria) and centrifuged at 3500 rpm for 10 min. Supernatants were transferred in 1-mL Eppendorf tubes and subsequently frozen at − 80 °C for long-term storage (until SERS analysis). For HCC, patients’ samples were collected at the time of diagnosis before any treatment.

### SERS substrate fabrication

The plasmonic paper substrates in use were fabricated according to an in-house developed procedure, following a dip-coating of filter paper with citrate-reduced silver nanoparticles [18]. The synthesis of the colloidal nanoparticles follows the recipe of Lee and Meisel [19]. Briefly, 10 mL of sodium citrate 1.1% w/w has been added dropwise to 500 mL boiling solution of AgNO_3_ 1.1 mM under magnetic stirring for 1 h and kept at dark. All glassware used for this synthesis was previously cleaned with nitric acid and Nochromix solutions (GODAX Labs Inc.), and thoroughly rinsed with Milli-Q water after each cleaning step. The resulting nanoparticles have been concentrated 10 times in volume with an ultra-centrifuge (60 min at 45000 rpm). Afterward, 1 cm^2^ filter paper squares were placed well-wise in a 24 multi-well plates with 3 mL of the concentrated Ag colloid. The addition of 62 μL of 1 M sodium citrate tribasic allowed NP aggregation and precipitation on the paper. After 7 days of incubation, the supernatant was removed and the substrates were transferred and stored in Milli-Q water, in dark and at room temperature, until use. The substrates prepared as described were stable for 3 months.

### SERS instrumentation

The spectra collection has been performed in air at room temperature with an i-Raman Plus portable system (BWS465-785S) through a compatible Raman video microscope (BAC151B) and with the BWSpec software (version 4.03_23_c), by B&W Tek (Newark, DE). Excitation was obtained with a 785-nm laser with an output power of about 400 mW. Laser light delivery to the sample and scattering collection occurred through an optical fiber probe connected to a compatible Raman video microscope. The instrument spectrograph had an average spectral resolution of 2.4 cm^−1^. The laser spot diameter at the sample was of 105 μm, obtained by using a × 20 Olympus objective (N.A. 0.25, working distance 8.8 mm). Spectra collection was performed with a single accumulation of 10 s CCD exposure, and with a laser power at the sample of 38 mW (10% of the maximum laser output). Using these experimental conditions, no substrate photo-degradation was reported. Paracetamol samples were used as standard reference samples during every measurement session to check spectrometer wavelength calibration.

### Sample preparation and SERS measurement

Serum samples were immediately analyzed after thawing. Five microliter drops of serum were dropped on the surface of the plasmonic paper substrates and let dry for 20 min. Later, the plasmonic paper substrates were placed under the i-Raman plus portable microscope objective on a glass microscope slide, and spectra were collected at room temperature (25 °C) in three technical replicas for each sample, which were averaged before further preprocessing and analysis. Data was collected on 5 different days and over 3 different batches of substrates. Sample collection was stratified over the different batches of substrates and over various days, so that on each day, an equal number of samples from both H0T and CTR classes and from each substrate batch was measured. This way, differences observed between classes cannot be related to the measurement day or to the substrate batch used. Also, measurements were randomly collected by two different operators.

### Data preprocessing, analysis, and visualization

Spectra have been entirely processed using the R environment for data analysis [20]—version 3.6.2 (2019-12-12). In particular, the package *hyperSpec* [21] was used for data import and visualization. The preprocessing steps included (i) Raman shift range selection (400 to 1800 cm^−1^) and data interpolation by local polynomial regression fitting (loess) to a new wavelength axis with a spacing of 2 cm^−1^, (ii) baseline correction (package *baseline* [22], method *modpolyfit*, polynomial degree = 4), (iii) vector normalization. Examples of baselines are shown in Fig. S1 of the ESM. After baseline correction, the Raman shift range was further cropped from 430 to 1730 cm^−1^ to delete possible artifacts due to the baseline subtraction present at the borders of the spectral range. A PCA-LDA prediction algorithm was used, in which a number of principal components (PC) were selected for a linear discriminant analysis (LDA). Principal components analysis (PCA) was performed using the *prcomp* function, centering but not scaling data. The cumulative proportion of explained Variance for the first 20 principal components of the dataset is available as ESM (Fig. S2A). The function *lda* from the *MASS* package [23] was used for the LDA. A RDCV [10] was chosen as validation strategy, in which the number of PC to be used in each LDA model was iteratively optimized using independent portions of the dataset in an “inner k-fold cross-validation loop” (*k* = 7), while an “outer k-fold cross-validation loop” (*k* = 3) is used to cross-validate the optimized models on independent folds of the dataset. Data partitions in both loops were created using the *createFolds* function of the *caret* package [24]. Data partition was stratified, so that each fold contained the same proportions of the classes considered. Note that the PCA was performed for each loop only for the train set, so that train and test sets were kept well separated and no information from the test set was introduced in the PCA-LDA model. The double cross-validation was repeated *n* times (*n* = 100), generating 300 optimized partial models (each from *k*-1 folds). For the RDCV, functions were also used from packages *chemometrics* [25], e1071 [26], and *ROCR* [27].

Confusion matrices were obtained for each of the 100 repetitions of the cross-validation by summing the partial confusion matrices of each fold. Quality performance metrics (sensitivity; specificity; accuracy; PPV—positive predicted values; NPV—negative predicted values; and AUC—area under the curve) for each repetition were calculated then from these confusion matrices, yielding a distribution of 100 values for each metric. The confidence intervals (95%) for sensitivity, specificity, accuracy, PPV, and NPV were calculated using the *binom.confint* function of the *binom* package [28], assuming binomial distributions. ROC curves for each repetition were generated by summing the prediction probabilities of each fold obtained with the *ROCR* package [27]. The confidence intervals for the AUC were calculated using the *cvAUC* package [29], according to LeDell et al. [30].

All figures were prepared using the R environment for data analysis [20]. Boxplots have been produced using the *ggplot2* [31] package, and the *ggsignif* [32] package was used to calculate and display significant differences between distributions.

## Results and discussion

**Fig. 1:**
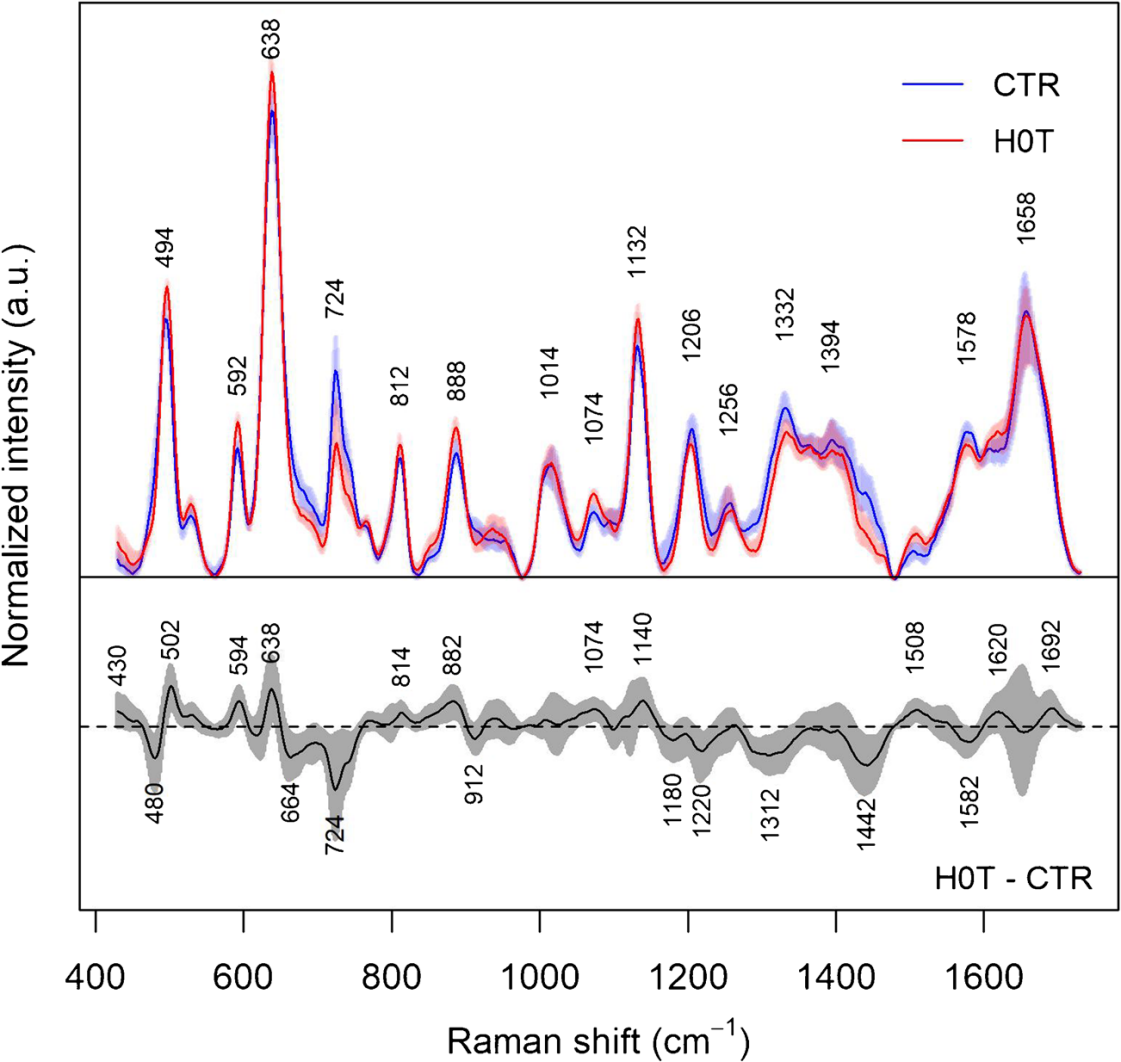
Comparison between the medians of SERS spectra of serum from H0T (*n* = 72) and CTR groups (*n* = 72). Interquartile ranges of the SERS intensity for the two groups are shown as shaded areas. Medians and interquartile ranges were calculated from intensity normalized spectra. The intensity difference between H0T and CTR is reported in the lower part of the figure

Median SERS spectra of serum from the two classes considered, i.e., patients diagnosed with hepatocellular carcinoma (H0T) and controls (CTR) are reported in Fig. 1, along with the median difference spectrum. For the first time, a large dataset of SERS spectra of serum has been collected using Ag “plasmonic paper” substrates, i.e., paper coated with Ag nanoparticles, previously described and characterized by our group [18]. The spectra in Fig. 1 display the characteristic purine bands of label-free SERS of serum and plasma previously reported for other substrates [5]. The main advantage of using such paper-based solid substrates, with respect to colloidal substrates, is that an intense SERS spectrum can be rapidly obtained without the need to de-proteinize serum samples to promote aggregation [33], as the nanoparticles on the plasmonic paper are already aggregated. Simply depositing few microliters of serum directly on the plasmonic paper, without the need of any sample preprocessing or mixing with metal colloids, allows the collection of an intense SERS spectrum. The SERS spectra in Fig. 1 present some similarities with those recently reported from plasma on a slightly different plasmonic paper [34], where purine bands still dominate the spectrum. As SERS spectra of plasma and serum are not expected to show marked differences [33], the differences between these two spectral datasets could be due to still unknown differences in the physicochemical characteristics of the two surfaces (arising from different preparation protocols), or perhaps to the different population characteristics of the subjects involved in the study (only obese subjects were enrolled for the other study).

SERS spectra from the two classes present some dissimilarities, as evidenced by the difference spectrum represented in the lower part of Fig. 1. A cursory inspection of positive and negative bands in the difference spectrum suggests that uric acid [33] (positive bands at 594, 638, 812, 888, and 1132 cm^−1^) is relatively more abundant in the sera of HCC patients than in those of controls, whereas hypoxanthine [33] (negative bands at 724 cm^−1^), ergothioneine [35] (negative bands at 480, 1220, 1442, 1582 cm^−1^), and perhaps glutathione [36] (negative bands at 664 and 912 cm^−1^) are relatively less abundant in HCC patients.

Similar differences involving an increase in the uric acid-hypoxanthine SERS band intensity ratio were reported for liver diseases in general by Shao et al. [37], and more specifically for different fatty liver stages (NASH vs. NAFL) in a recent paper by our group [34]. As hypoxanthine is ultimately converted to uric acid by xanthine oxidase, these reports seem to suggest a role of the purine metabolism, and in particular of xanthine oxidase, as a general marker for liver function. Such conjecture has been recently supported by other papers as well [38].

On the other hand, a relative decrease in the SERS intensity of ergothioneine bands for liver cancer patients with respect to controls has been also reported (although with a different band interpretation) by Xiao et al. [39] and Liu et al. [40]. Ergothioneine, a natural amino acid that we assume with the diet and that has been found in high concentrations in the liver [41], appears to be often observed in SERS spectra of various biofluids, including serum and plasma [35]. Although its role is still not known, one of the most often cited hypotheses is its possible role as a potent antioxidant [41]. The fact that bands tentatively assigned to glutathione were also found to be less intense in HCC patients, consistently with those of ergothioneine, indicates a different oxidative status in HCC patients compared to controls. Interestingly, oxidative stress has been indeed suggested to play a relevant role in liver carcinogenesis from different etiologies [42].

Building upon these spectral differences, a predictive model can be trained to classify SERS spectra of serum collected on plasmonic paper as belonging to subjects with (i.e., positive class, labeled as H0T) or without HCC (i.e., negative class or controls, labeled as CTR). A RDCV strategy [10] has been adopted to optimize and evaluate the performance of a PCA-LDA model to classify the SERS spectra of serum.

The RDCV generated optimized models differing one another by the composition of train and test segments for the outer RDCV loop, and for the number of PC used in the LDA algorithm, as resulting from an optimization independently performed in the inner RDCV loop. RDCV is structured so that each optimization and validation step is performed on an independent test set. Thus, overfitting is avoided by each model, since no information from the test set is used to build the model used to predict it.

The only information needed as external input is the maximum number of PC to be considered for the inner loop. In this study, the maximum number of PC for the optimization loop was set to 7, as the first 7 PC calculated from the PCA of the entire dataset explained up to 90% of the spectral variance (see Fig. S2A in the ESM). A visual inspection of the loadings of PC7 (Fig. S2B in ESM) indicates that relevant spectral information is still present, ruling out the possibility of including just noise.

**Fig. 2:**
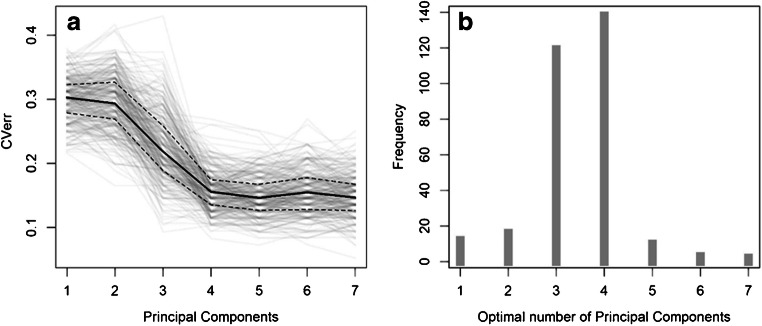
Characterization of the PCA-LDA models produced by the RDCV. **a** Curves for the inner cycle of the RDCV, showing the cross-validation error (CVerr) when using a different number of PC. **b** Frequency plot for optimized models, showing the number of models generated (i.e., frequency) using a specific number of PC, as a consequence of model optimization

In the inner loop, the optimal number of PC is chosen by applying the so-called one-standard-error rule [6, 43]. The cross-validation error curves for all the models obtained by the RDCV are reported in Fig. 2a, showing that the models do not improve by including more than 4 PC. Consistently with this picture, Fig. 2b shows that most of the models were optimal when up to 3 or 4 PC were included as variables for the LDA, whereas a negligible fraction retained more than 4 PC. These results are suggesting that the PC after the 4th are not meaningful in differentiating between the two classes, although we still do not know which ones, among the first four, are the most relevant.

In the studies reported so far dealing with the classification of label-free SERS spectra of biofluids [3, 5], the value of the parameter for the classification algorithm (e.g., number of PCs or latent variables for PCA-LDA or PLS-DA) was arbitrarily selected on the basis of the information available from the whole dataset (e.g., the cumulative variance explained by a PC or the *p* value for a statistical test); this was not the case for RDCV. Conversely, in the present study, the use of a RDCV ensured an automated parameter selection for each model based on cross-validation, without using any information from the spectra to be predicted in the outer loop, thus avoiding the risk of overfitting during model optimization.

**Fig. 3:**
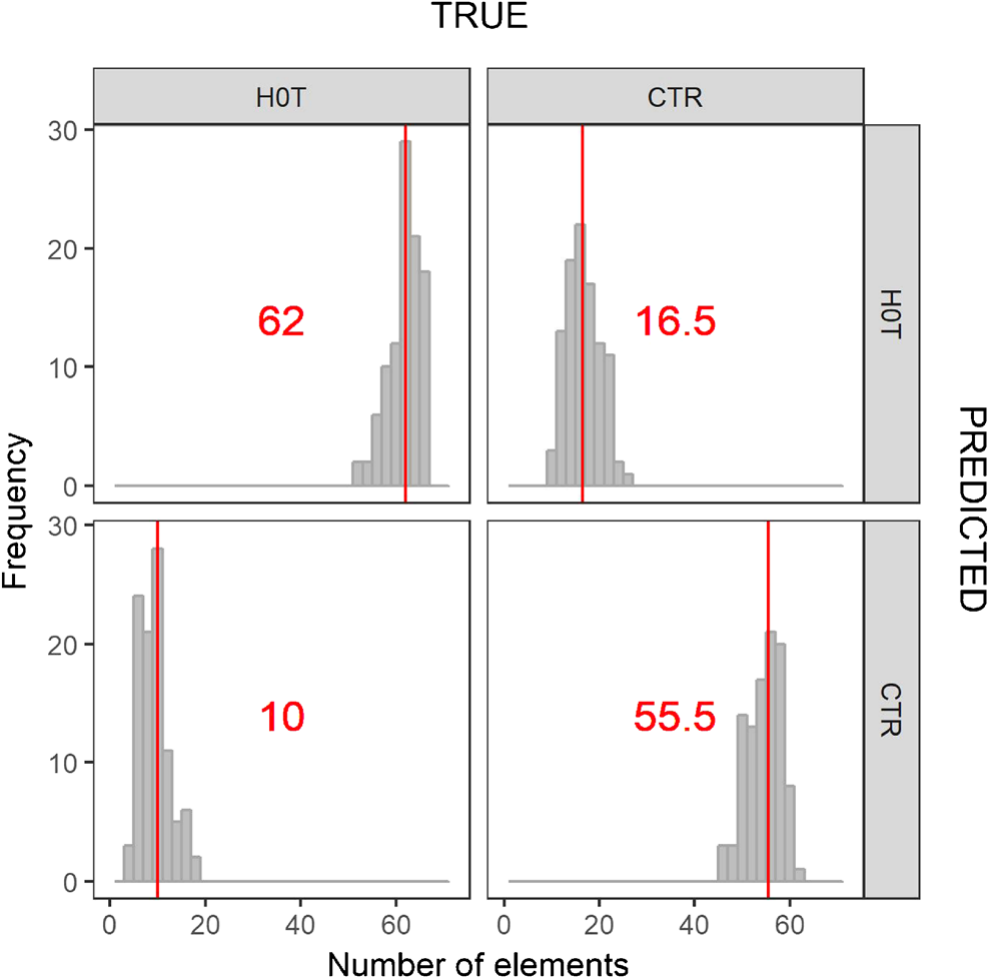
Statistics for the confusion matrices resulting from the predictions of the RDCV optimized models. Median values are shown in red

The performance of each optimized model generated by the RDCV was validated in the outer RDCV loop by comparing the predictions to the actual classes of an independent test set. Each iteration of the RDCV produced a confusion matrix (also known as error matrix), and the statistics of all the confusion matrices thus obtained is summarized in Fig. 3. The medians of the distributions for true positive, true negative, false positive, and false negative values give a first estimation on the overall performance of the PCA-LDA algorithm when up to 4 PCs are used (e.g., on a total of 72 spectra from sera of HCC patients, 62 are correctly predicted while 10 are misclassified as controls).

FOM such as accuracy, sensitivity, and specificity were calculated from the confusion matrix of each optimized model, yielding a distribution of values for each FOM from which confidence intervals were calculated (Table 2). Being able to estimate confidence intervals for these FOM is another advantage of using a repeated cross-validation strategy, as it allows an uncertainty estimation of the predictive capabilities of the model.

**Table 2:** Figures of merit calculated from the optimized models generated from the RDCV

Figure of merit	Average (95% CI)
Accuracy	81.1 (74.7–87.3)
Sensitivity	85.9 (77.8–93.4)
Specificity	75.9 (66.1–85.4)
PPV	78.2 (68.5–87.4)
NPV	84.3 (74.6–93.3)
AUC	87.6 (87.0–88.2)

**Fig. 4:**
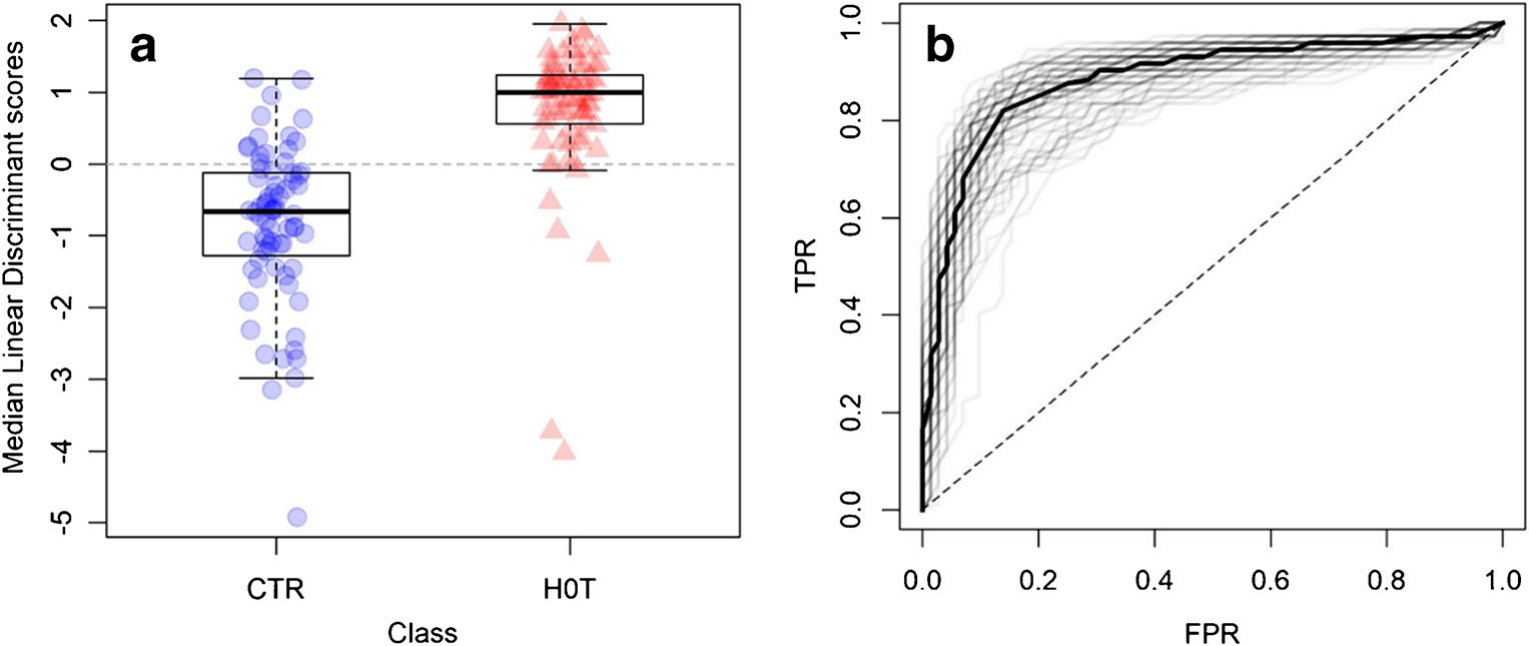
Medians of the LD scores (**a**) for each sample, calculated over the optimized models from the RDCV; ROC curves (**b**) of the optimized models from the RDCV. The average ROC is shown as non-transparent, black trace

The average FOM and the corresponding confidence intervals suggest that a PCA-LDA model can distinguish between the two groups with an overall accuracy of about 80%, favoring model sensitivity (86%) at the expenses of specificity (76%). Another perspective on the performance of the PCA-LDA model can be gained by inspecting the LD scores (Fig. 4a) and the ROC curves (Fig. 4b) for each model generated by the RDCV. To further assess the statistical significance of these results, they were compared to those obtained from a validation of permuted data (i.e., permutation test [12]) in which the class labels were randomly assigned (Fig. S3 in the ESM). The permutation test confirmed the significance of the results obtained from the RDCV validation from the dataset with the correct class labels.

**Fig. 5:**
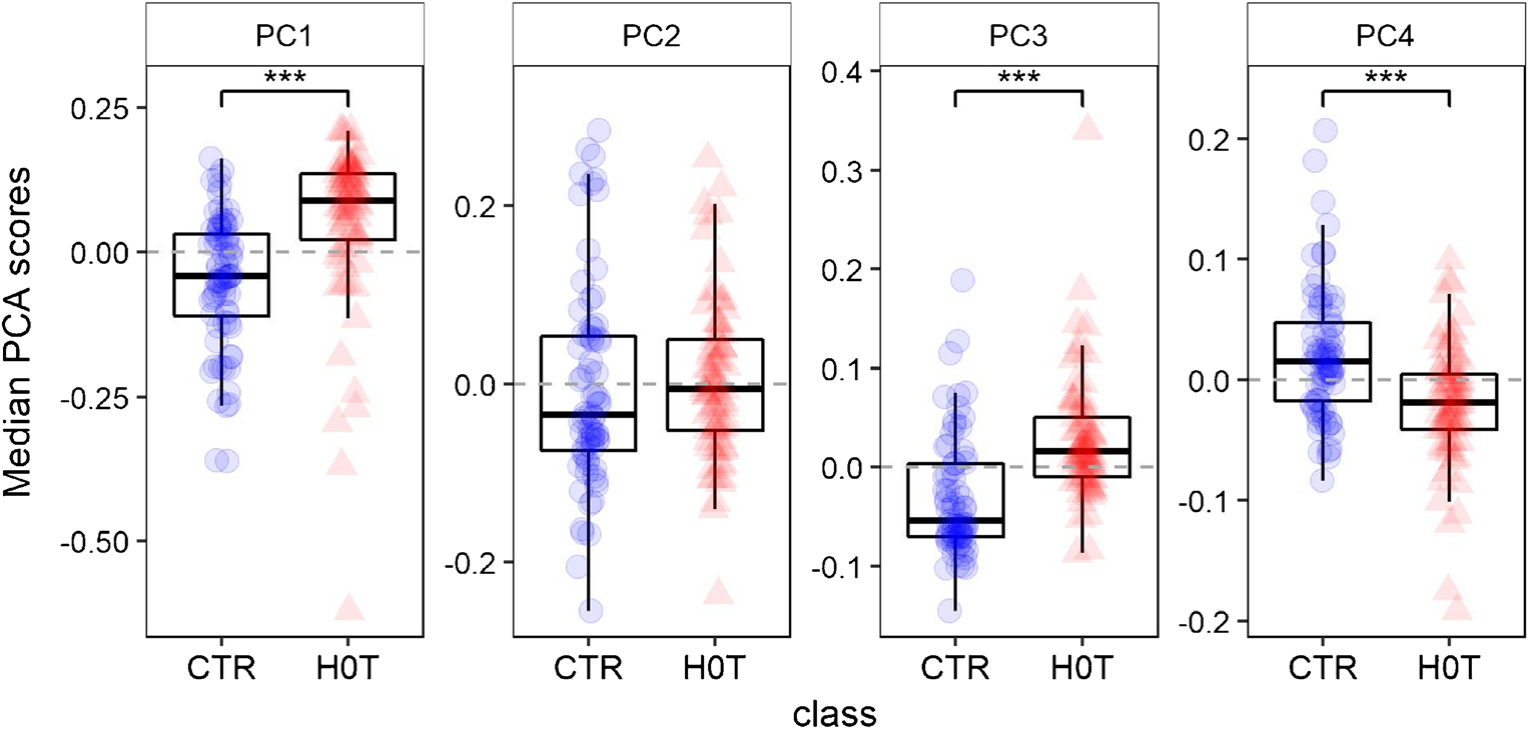
Medians of the PCA scores for the first 4 principal components, grouped according to class, calculated over the optimized models from the RDCV; the significance with respect to the Mann-Whitney *U* test for the 2 classes is reported for each component

While an analysis of the optimized models (Fig. 2) illustrates the most frequent number of optimal PC (i.e., 3 or 4), it does not provide information about which components are most important for the performance of the PCA-LDA model. This information can be gained by looking at the medians of the PCA scores for each class, for each PC (Fig. 5). While PC 1, 3, and 4 all seems to be useful to distinguish between the two classes, the second PC seems to be irrelevant.

**Fig. 6:**
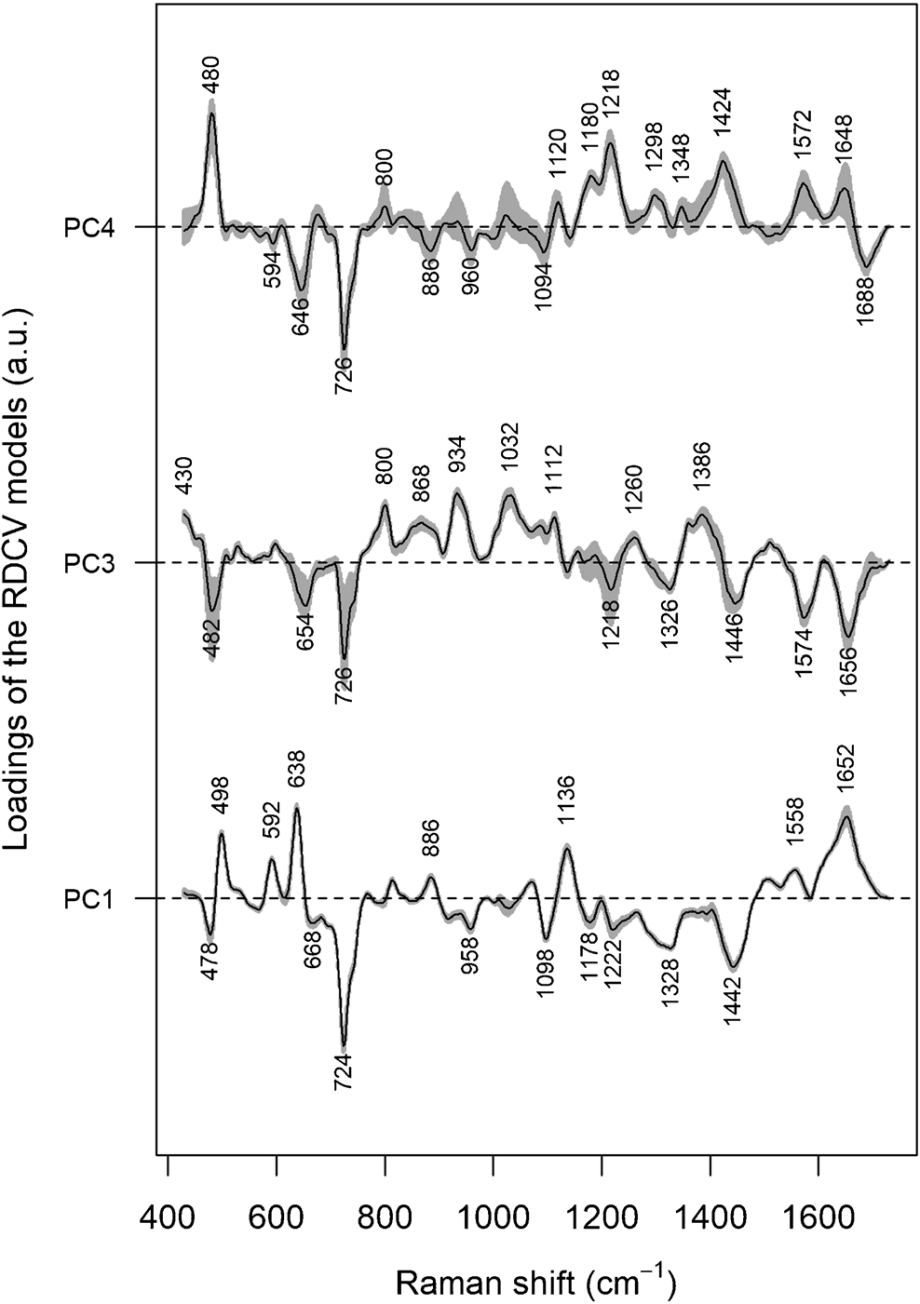
Medians of the loadings for principal components 1, 3, and 4, calculated over the optimized models from the RDCV; interquartile ranges are reported in gray

The question arises about a biochemical interpretation of these PC: what metabolites allow the distinction between the two groups? An inspection of the loadings of PC 1, 3, and 4 (Fig. 6) can help in answering this question, by interpreting the loadings in terms of spectral bands. The negative peaks in the loadings of PC1 can be interpreted as hypoxanthine bands, whereas the positive loadings seem to be correlated with the uric acid bands, corroborating the impression that these two metabolites are important in discriminating between the two groups (with uric acid relatively more abundant and hypoxanthine less abundant in the H0T group). The positive peaks in the loadings of PC3 are less easy to interpret than those of PC1, but negative peaks can be interpreted as bands due to hypoxanthine, ergothioneine, and (tentatively) glutathione, confirming the role of these substances in distinguishing between H0T and CTR classes, being relatively less abundant in the H0T class. Ergothioneine bands (especially the intense band at 480 cm^−1^) can be also identified in the positive peaks of the PC4 loadings, endorsing the hypothesis that this metabolite is relatively more abundant in the CTR group. In general, the information in Figs. 5 and 6 is corroborating the picture given by Fig. 1, suggesting that the PCA-LDA models are indeed using these spectral differences to discriminate between classes.

The possibility of checking the workings of the classification model in terms of biochemical information given by the loadings is a further advantage of the PCA-LDA (but also of the PLS-LDA) models with respect to other less transparent algorithms (e.g., non-linear models such as support vector machines [10]) working more like “black boxes” concerning spectral interpretation. The fact that the model is based on real spectral differences (and not just noise or artifacts) is an indication that overfitting is less likely, while a biochemical interpretation of the differences used by the model can be exploited to gain a better insight into the biochemistry of the disease.

The clinical relevance of uric acid in relation to cancer risk, recurrence, and mortality has been suggested since many years [44] and it has been extensively reviewed, among others, by Fini et al. in 2012 [45], and more recently by Battelli et al. [46]. The association of hyperuricemia with cancer occurrence and recurrence has been reported in various cancer types, including HCC. More recently, high levels of serum uric acid were specifically suggested as risk factor for recurrence of HCC [47]. Hypoxanthine is metabolically related to uric acid via the xanthine oxidoreductase enzyme. Unfortunately, no information exists about a possible correlation of ergothioneine to HCC or to any cancer. However, we consider this as an interesting finding that has to be further explored in relation to HCC, especially when considering the antioxidant potential of this molecule. The unbalanced redox state is one of the drivers of hepatic carcinogenesis, as oxidative stress induces genomic damage and genetic instability leading to mutations.

## Conclusions

Label-free SERS spectra of whole serum can be rapidly obtained from Ag plasmonic paper substrates. Spectra from the HCC and CTR groups showed consistent differences, which were exploited by the PCA-LDA models for classification purposes, with satisfying results in terms of performance. The use of a RDCV approach for the PCA-LDA applied to label-free SERS data allowed to automatically determine the number of PC to be used in LDA, and to calculate confidence intervals for FOM. Most importantly, the analysis of the RDCV results allowed to pinpoint which are the most relevant PC for the LDA model, and to interpret their loadings in terms of metabolites. This analysis confirmed that uric acid, hypoxanthine, ergothioneine and possibly glutathione, which were responsible for most spectral differences observed, have been effectively used by the PCA-LDA algorithm for classification. These metabolites are thus possible candidates as HCC markers, and might be investigated in further studies.

## Supplementary information

ESM 1 (PDF 768 kb).

## Data Availability

The dataset consisting of all spectra is available for download on Zenodo (zenodo.org), DOI: 10.5281/zenodo.4277797.
