# Supplementary material for: Repeated double cross-validation applied to the PCA-LDA classification of SERS spectra: a case study with serum samples from hepatocellular carcinoma patients
Source: Anal Bioanal Chem. 2020 Dec 8;413(5):1303–12. doi: 10.1007/s00216-020-03093-7 (PMC7892523; doi:10.1007/s00216-020-03093-7)
Supplement: Supplementary file 1 — (PDF 768 kb). [file 216_2020_3093_MOESM1_ESM.pdf]

SUPPLEMENTARY INFORMATION

Repeated double cross-validation applied to the PCA-LDA classification of SERS spectra: a case study with serum samples from hepatocellular carcinoma patients

Elisa Gurian (1), Alessia Di Silvestre (1), Elisa Mitri (1), Devis Pascut (2), Claudio Tiribelli (2), Mauro Giuffrè (3) (4), Lory Saveria Crocè (2) (3) (4), Valter Sergo (1) and Alois Bonifacio (1)

AFFILIATIONS

(1) Raman Spectroscopy Lab, Dipartimento di Ingegneria e Architettura (DIA), University of Trieste, via Valerio 6, 34127, Trieste (TS), Italy

(2) Fondazione Italiana Fegato – ONLUS, Area Science Park, SS14, km163.5, 34149, Basovizza, Trieste (TS), Italy.

(3) Department of Medical Sciences, University of Trieste, Strada di Fiume, 447, 34129 Trieste, Italy.

(4) Clinica Patologie Fegato, Azienda Sanitaria Universitaria Giuliano Isontina (ASUGI), Via Costantino Costantinides 2, Trieste, Italy.

	HCC (n= 72)	Healthy (n=72)
**Patients characteristics**		
**Age (mean, 95%CI)**	69 (67-71)	56 (55-57)
**Etiology**		
Alcohol metabolic	44	
Alcohol metabolic viral	13	
Viral	10	
Other*	5	
**Disease scores**		
CTP A/B/C	50/17/3	
BCLC 0/A/B/C-D	7/38/21/6	
**Tumor parameters**		
**Number of lesions**		
Single ≤ 2cm	9	
Single or 3 ≤3cm	21	
Large-single or multi	41	
**Alpha-fetoprotein**		
<20 ng/mL	37	
20 - 400 ng/mL	8	
>400 ng/mL	9	

Table S1 Detailed characteristics of the study population.

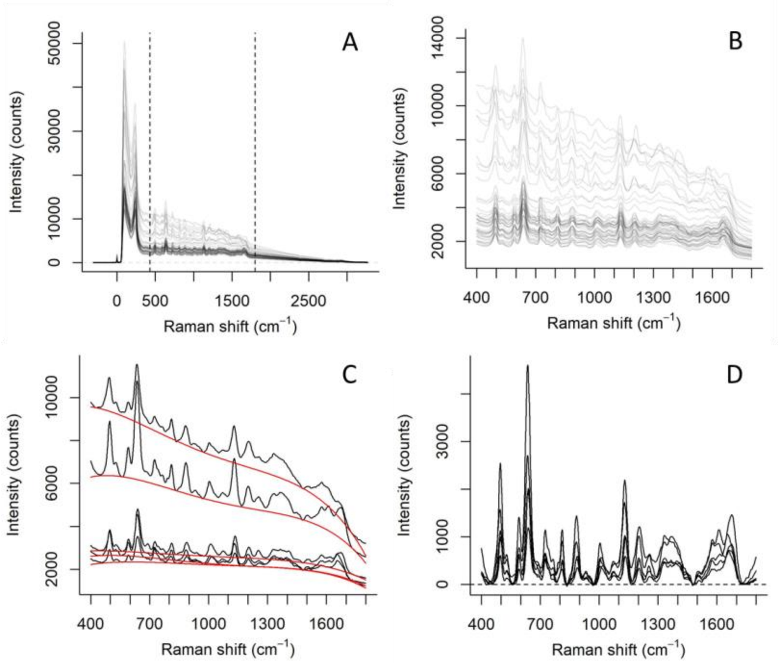

Fig. S1 Spectral preprocessing: (A) un-processed data and spectral region used for cropping; (B) data after cropping; (C) examples of spectra before baseline subtraction, with calculated baseline reported as red lines; (D) the same spectra shown in (C) after baseline subtraction.

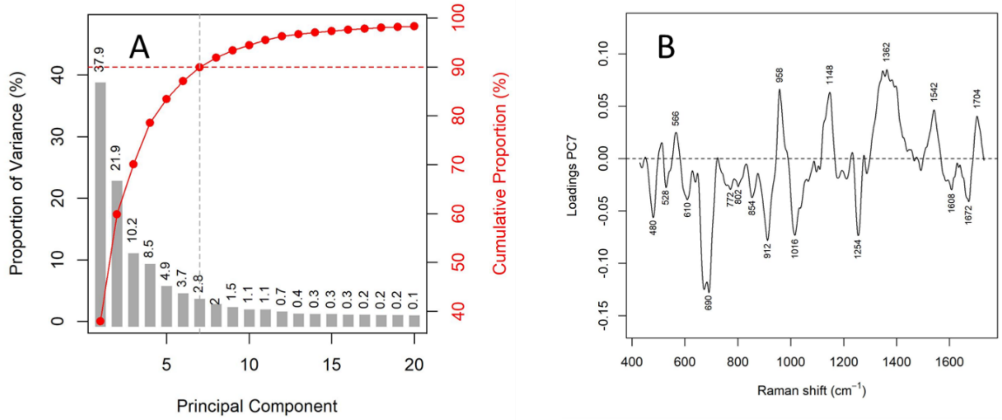

Fig. S2 Cumulative proportion of explained Variance (A) for the first 20 principal components of the dataset, together with the loadings for the PC7, since the first 7 PC explain up to 90% of the spectral variance.

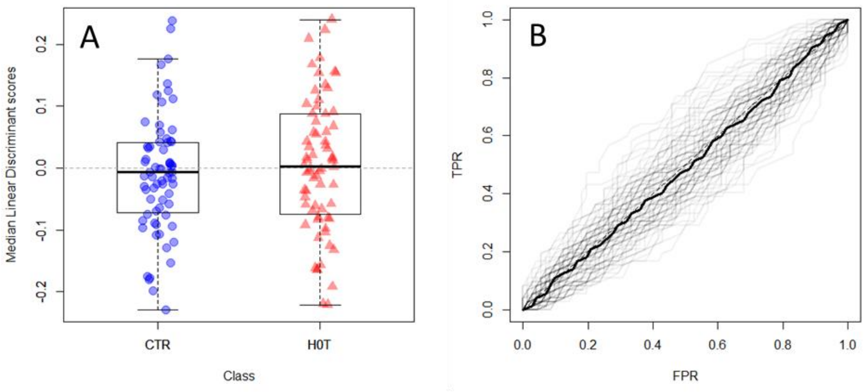

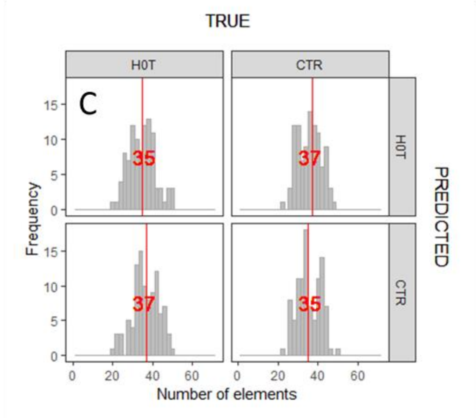

Figure of merit	Average (95% C.I.)
Accuracy	49.3 (41.2 – 57.4)
Sensitivity	48.7 (37.3 – 60.0)
Specificity	49.8 (38.5 – 61.2)
PPV	49.1 (35.4 – 63.0)
NPV	49.4 (35.8 – 62.9)
AUC	50.3 (46.4 – 51.3)

Fig. S3 Results (corresponding to figures 3 and 4, and to table 2 or the manuscript) of a permutation test (with 100 permutations) for the optimized PCA-LDA model (i.e. n°PC = 4), in which the class labels were randomly shuffled (i.e. each sample has a class label randomly chosen). Results are those expected from pure chance (i.e. “zero hypothesis” H0 classification), with all Figures of Merit close to 50%, markedly different from the results obtained from the dataset with the correct class labels. In particular: Figures of Merit (bottom right) calculated from all the permutations; medians of the LD scores (A) for each sample, calculated over the different permutations; ROC curves (B) of all permutations (the average ROC is shown as non-transparent, black trace); (C) statistics for the Confusion Matrices resulting from the permutations (median values are shown in red).
